# The inverse relationship between blood amylase and insulin levels in pigs during development, bariatric surgery, and intravenous infusion of amylase

**DOI:** 10.1371/journal.pone.0198672

**Published:** 2018-06-06

**Authors:** Kateryna Goncharova Pierzynowska, Liudmyla Lozinska, Jarosław Woliński, Stefan Pierzynowski

**Affiliations:** 1 Department of Biology, Lund University, Lund, Sweden; 2 Anara AB, Trelleborg, Sweden; 3 PROF, Lublin, Poland; 4 Department of Biochemistry and Biotechnology, Vassyl Stefanyk Precarpathian National University, Ivano-Frankivsk, Ukraine; 5 Department of Animal Physiology, The Kielanowski Institute of Animal Physiology and Nutrition, Polish Academy of Sciences, Jabłonna, Poland; 6 Department of Biology, Institute Rural Medicine, Lublin, Poland; Medical University of Vienna, AUSTRIA

## Abstract

The purpose of this research is to explore the link between plasma amylase and insulin levels in growing pigs. Blood was obtained from piglets ranging in age from preterm (8 days to full gestation period), up to postnatal day 90 (2 months post-weaning) that underwent either duodenal-jejunal bariatric interposition surgery or a sham-operation. Plasma amylase activities in preterm and full-term neonates ranged between 500–600 U/L and were decreased by 50% two months post-weaning. Preprandial insulin and C-peptide levels in neonate piglets were not detectable, however they rose gradually after weaning. An increase in plasma amylase activity was observed in the young pigs that underwent duodenal-jejunum bypass (metabolic) surgery. The increase in blood pancreatic amylase activity after an intravenous amylase infusion lowered the subsequent glucose-stimulated insulin/C-peptide release. We suggest a role for blood amylase in the regulation of glucose homeostasis after observing high blood amylase levels in neonate pigs, in pigs that underwent metabolic surgery, and as a result of the reduced glucose-stimulated insulin response following intravenous amylase administration. Blood amylase level is a dynamic physiological parameter, which is not merely a consequence of exocrine pancreatic digestive enzyme production, but rather a regulated factor involved in glucose assimilation and prandial insulin regulation.

## Introduction

Approximately 100 years ago, researchers discovered the presence of pancreatic enzymes, such as amylase, in the blood [1–3]. Serum amylase (diastase) levels were found to be associated with various physiological changes, such as impaired hepatic function, and not merely a marker of exocrine pancreatic function [2, 4]. Serum amylase activity was found to be highly variable during the early stage of the neonatal period in humans, usually close to or below the lower limits of normal established for adults [5]. More recently, higher blood amylase levels were positively correlated with higher insulin sensitivity, while lower plasma amylase activities were observed in patients with obesity and metabolic disorders [6–9]. All of these indicate the potential influence of blood amylase on the functionality of the endocrine pancreatic hormones.

Nevertheless, the discovery of a halo phenomenon for many years added support for the oversight function of pancreatic hormones over acini cells activity and enzyme production [10]. The theoretical separation between the exocrine and endocrine pancreas was accepted as an undiscussed dogma, and as a result, investigations into the meaning and physiological meaning of amylase circulation in the blood dramatically decreased.

Previous conflicting data regarding the source of blood amylase levels has added confusion to subsequent investigations which till now remain unsolved. In the early 1930s, removal of the pancreas and salivary glands in a rat model did not cause a significant decrease in blood amylase [11]. Together with the hypothesis that the liver might also be involved in the maintenance of blood amylase levels, in 1958, Dreiling suggested that the mechanism that regulates blood amylase level could even be extra-pancreatic [2, 12].

In current medicinal practice, a significant increase in serum amylase levels (together with lipase) is used as a marker for injury to the exocrine pancreas (*acute* pancreatitis, pancreatic cancer). However, the use of increased blood amylase levels as a marker for *chronic* pancreatitis was considered not to be specific enough [13–15]. Numerous patients without gastroenterological symptoms could present with variations in blood amylase levels. Nevertheless, even though the exocrine pancreas is one of the main sources of plasma amylase activity, and serum pancreatic amylase level provides some information regarding pancreatic amylase production, the milestone statement that pancreatic blood amylase is a consequence of the “leakage” of pancreatic amylase into the blood requires more investigation. Is this “leakage” of pancreatic amylase into the blood regulated? We believe, that the physiological reason for the presence of circulating amylase in the blood deserves additional attention, independently of its source, first of all due to amylase’s potential involvement in carbohydrate metabolism regulation [2, 12, 16].

The connection between the exocrine and endocrine pancreatic function is evident in pancreatogenic diabetes and metabolic disorders. The best known treatments for diabetes nowadays are metabolic surgeries which involve exclusion of the duodenum [17], which could potentionally influence exocrine pancreatic function. Our recent results show that in the bypassed gut, the spatial separation of pancreatic enzymes from their substrates (food) reduces the involvement of alpha amylase in the digestive process and reveals amylase’s extra-digestive activity which correlates with improved prandial glucose tolerance [16, 18, 19]. Enteral administration of amylase was found directly influence glucose disposal in pigs [18]. The pig is commonly accepted as an appropriate model for the study of gastro-intestinal function in humans [20, 21]. Due to similarities in morphology and physiology of the gastrointestinal systems, enzymatic and hormonal factors, ingesta transit times and digestive efficiencies, pigs which undergo bariatric surgery could serve as appropriate models [20, 21] for studying the relationship between plasma amylase levels and glucose utilisation. Considering all of the above, the current study was designed to elucidate and emphasize the relationship between blood amylase and insulin levels. Thus highlighting the role of blood amylase in the intra-pancreatic regulation of the post-prandial insulin response, using a pig model.

## Materials and methods

The data presented was collected from: i) 18 non-suckled preterm piglets delivered via Caesarean section; ii) 4 full-term suckled piglets; iii) 3 pigs after duodenal-jejunal bypass surgery (DJB) and 3 sham-operated (SO) littermates that were monitored up until 3 months of age; iv) 3 pigs weaned at 4 weeks of age and monitored up until 3 months old were used to test an intravenous pancreatic amylase infusion. All pigs (Swedish Landrace x Yorkshire x Hampshire) were housed individually in separate pens (1.0 x 1.5 m), equipped with a water dispenser, sawdust for bedding and a heating lamp. The pens had windows to allow social interaction between neighbouring pigs. All experimental procedures were approved by the Malmö –Lund, Local Ethical Review Committee for Animal Experiments (Permit Numbers: M170-14, M181-15). All efforts were made to minimize animals’ suffering.

The weaned pigs were fed a standard cereal-based feed (“Morawski”, Żurawia, Poland or Växtill 320, Lantmännen, Sweden) corresponding to 4% of their body weight daily, half the ration was given in the morning, between 08:00–09:00, and the other half in the afternoon, between 16:00–16:30. The pigs had free access to water for the duration of the experiments.

DJB surgery was performed with the creation of a biliopancreatic limb (approx. 100 cm long, bypassed from the food), alimentary limb (approx. 100 cm long), and remaining common limb. Sham operated littermates underwent surgical manipulation without intestinal rearrangements as previously described [19].

Another three healthy, young, unoperated pigs were used to assess the effects of an intravenous amylase infusion on the outcome of an intravenous glucose tolerance test (Iv.GTT). Porcine pancreatic alpha-amylase (Lee Biosolutions, Inc., Maryland, USA), 5 000 U/ pig in sterile 0.9% NaCl, was administered twice via the jugular vein catheter, 1h and then 1 minute prior to performing the Iv.GTT. For the Iv.GTT, glucose was administered as a 40% solution (1g glucose/kg bwt) via the *jugular vein* catheter, which was flushed with 8 ml of 0.9% sterile saline solution immediately thereafter. Each pig underwent the Iv.GTT twice, with and without (negative control) the intravenous amylase infusion, following a Latin square design.

At the end of the study, all pigs were euthanized by an i.v. injection of an overdose of pentobarbital sodium (Allfatal Vet. Omnidea, Stockholm, Sweden, 100 mg/kg).

Blood samples collected from preterm and full-term pigs were obtained from the umbilical vein, around 1 hour following the C-section or natural birth. In all other cases blood was collected from an indwelling jugular vein catheter, inserted as previously described, a minimum of 1 week prior to test sample collection [19]. Blood samples were collected into BD Vacutainer^®^ glass Aprotinine K3EDTA tubes (BD Diagnostics, New Jersey, USA). Blood glucose concentrations were measured directly following blood sampling using a glucometer and test strips (Accu-Chek^®^ Aviva, Roche Diagnostics, Germany). The blood samples were immediately placed on ice before they were centrifuged at 3000 x g for 15 minutes at 4°C, and plasma was separated and stored at -20°C until further analysis.

Plasma insulin and C-peptide concentrations were measured using porcine insulin and porcine C-peptide ELISA kits (Mercodia, Uppsala, Sweden), according to the manufacturer's instructions. Plasma amylolytic activity was analysed using ethylidene-pNP-G7 (4,6-ethylidene-p-nitrophenyl-alpha, D-maltoheptaoside) as the substrate, according to the manufacturer’s instructions (Infinity Amylase Liquid Stable Reagent; Thermo Fisher Scientific, Middletown, Virginia, USA), with the modifications for a microtiter plate reader.

Data are expressed as mean ± standard error of the mean (SEM). The total area under the curve (AUC) was calculated for post-load blood glucose, insulin, and C-peptide levels, using the trapezoidal rule. All statistical analyses were carried out using the R (v. 3.0.1) programming environment. Insulin, C-peptide and amylase levels were compared using a paired (where appropriate) t-test at different times. We used a Bonferonni correction with α = 0.05 to correct for multiple testing where appropriate. In all statistical analyses p ≤ 0.05 was considered significant and p ≤ 0.1 was considered a tendency.

## Results

**The plasma amylase** activities were 539±17 U/L for preterm and 642±94 U/L for full-term, unsuckled neonate piglets, whereas plasma insulin levels were below the detectable limit in both groups of neonates (Table 1). Blood glucose levels in the first hours after delivery for preterm piglets were 3.0±0.2 mmol/L and 2.8±0.4 mmol/L in the full-term piglets at the preprandial stage. Healthy, weaned, 3 month old pigs showed significantly lowered plasma amylase activity (by 50%) compared to that observed in newborn, full-term piglets (p = 0.03, Table 1). The baseline preprandial insulin level was in the detectable range at 3.3 pmol/L in the adult pigs (Table 1).

**Table 1:** The inverse relationship between plasma amylase activity and plasma insulin concentration in pigs during maturation.

Plasma/Blood	Preterm newborn (n = 18)	Full-term newborn (n = 4)	1 month old, weaned (n = 3)	2 months old (n = 3)	3 months old (n = 3)
**Preprandial Glucose, mmol/L**	3.0±0.2	2.8±0.4	4.0±0.1	4.5±0.1	4.2±0.2
**Preprandial Insulin, pmol/L**	n.d.	n.d.	2.7±0.7	3.1±0.2	3.3±0.6
**Preprandial Amylase, U/L**	539±17 ^ab^	642±94 ^a^	490±107 ^ab^	440±82 ^ab^	309±11 ^b^
**Body weight, kg**	1.05±0.05	1.30±0.05	7.50±0.26	10.80±0.06	23.53±1.13

Data shown as mean ± sem. Bonferonni correction with α = 0.05 was used to correct data for multiple testing. Different superscripts within the same row indicate statistically significant differences between groups, p ≤ 0.05. n.d.—none detected.

**Fig 1:**
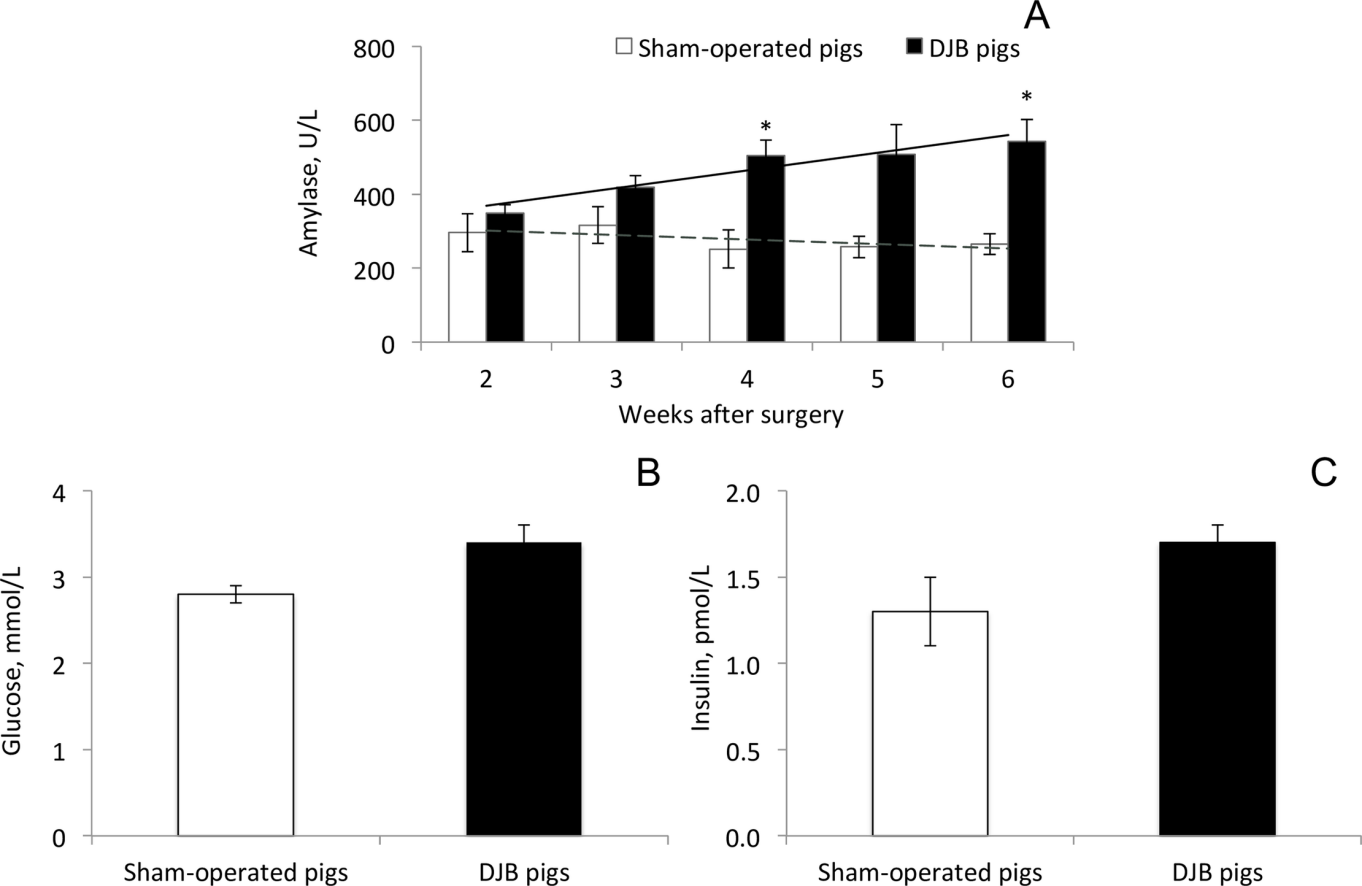
**A-C. Plasma amylase activity (A), blood glucose (B) and plasma insulin (C) concentrations in pigs that underwent duodenal-jejunal bypass (DJB) surgery compared to sham-operated littermates.** Data shown as mean ± sem (n = 3). Bonferonni correction with α = 0.05 was used to correct data for multiple testing. * p ≤ 0.05 significant difference between sham-operated and DJB pigs.

**Duodenal-jejunal bypass surgery** did not significantly affect plasma amylase activity in juvenile domestic pigs, 2 weeks following surgery (Fig 1A). However, a two-fold significant increase in blood amylase activity was observed compared to sham-operated littermates, 4 weeks following DJB surgery (p = 0.02, Fig 1A). Six weeks after the DJB surgery, baseline amylase activity tended to increase (p = 0.07) compared to the level observed at 2 weeks post-surgery in the DJB pigs, whereas it was unchanged in sham-operated littermates. There were no significant differences in fasting blood glucose concentration and fasting insulin concentration in DJB pigs, compared to that observed in sham-operated pigs (Fig 1B and 1C).

**Fig 2:**
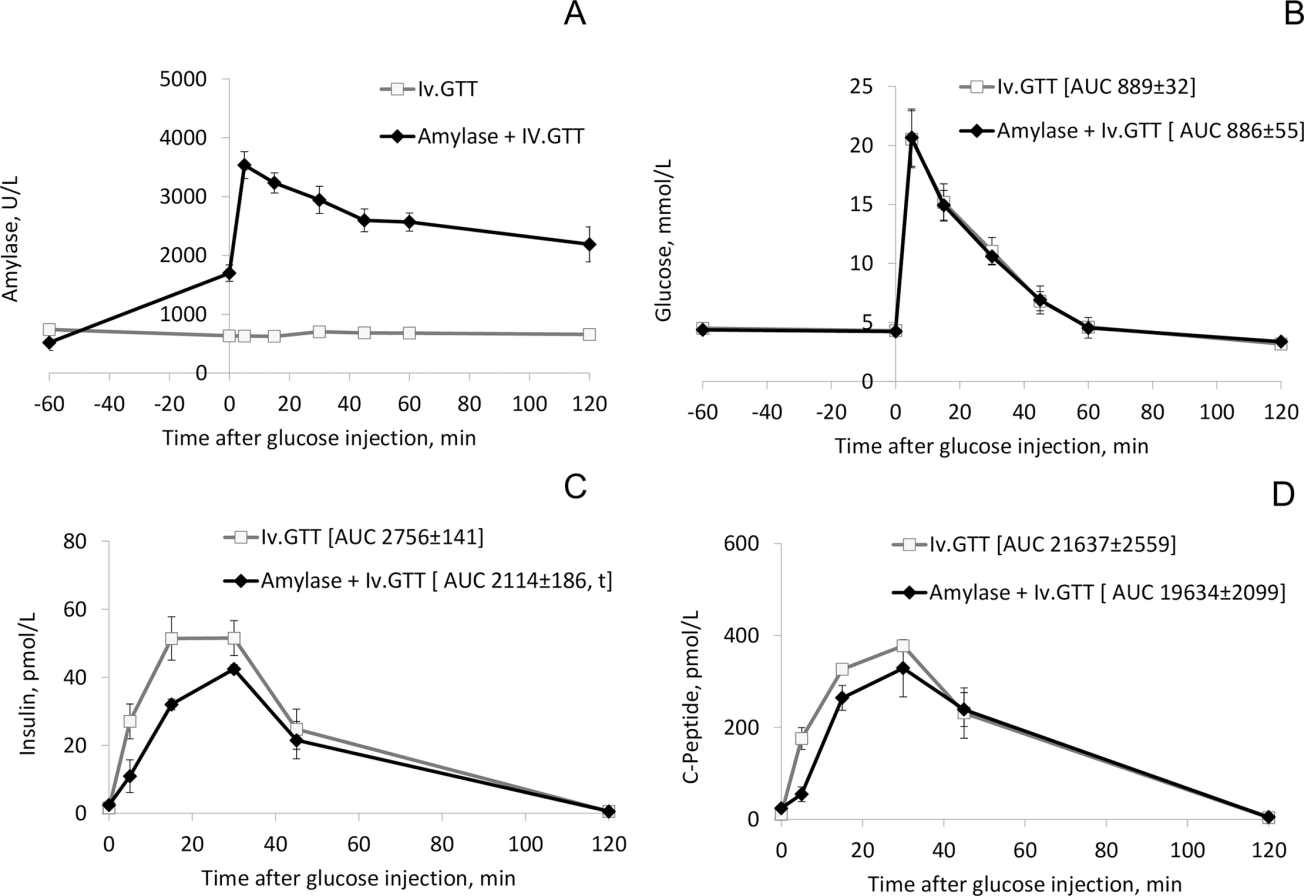
A-D. Plasma parameters in healthy unoperated growing pigs. (A) Amylase activity, (B) blood glucose, (C) plasma insulin, and (D) C-peptide concentrations during an intravenous glucose tolerance test (Iv.GTT) either alone or together with an intravenous pancreatic amylase infusion (Amylase + Iv.GTT). Data shown as mean ± sem (n = 3), with area under the curve (AUC) values shown in brackets, *t* indicates tendency with p ≤ 0.1. Data was not corrected for multiple testing.

**An intravenous infusion** of active pancreatic amylase, to pigs that were fasted overnight, significantly increased blood amylase activity level. Blood amylase levels were approximately 6-fold higher 5 minutes following the amylase infusions during the Iv.GTT, compared to baseline levels and compared to that observed at the corresponding time point without infusion of exogenous amylase (Fig 2A). Two hours following the amylase-glucose loading, plasma amylase levels were still as high as 60% of the level measured 5 min post loading. However, the increase in blood amylase activity, prior to the intravenous glucose challenge, did not affect the glucose curve (Fig 2B). At the same time, the amylase infusion significantly lowered the insulin response at 15 minutes (p = 0.04) post-infusion, compared to the control insulin response observed without amylase infusion (Fig 2C). The total area under the curve (AUC) for insulin response was significantly (p = 0.05) lower following the intravenous amylase administration. The intravenous amylase infusion also resulted in lowered C-peptide levels at 5 minutes (p = 0.01) following the amylase infusion during the Iv.GTT, compared to that obtained without amylase administration (Fig 2D).

The datasets generated and analysed during the current study are available at https://figshare.com/s/2507c43abc95d4b82791

## Discussion

Our observations reveal a new perspective for the presence of amylase in the circulation and its’ inverse association with insulin during maturation. Blood amylase could have some intra-pancreatic signalling effects which regulate endocrine pancreatic function. The specific interplay between insulin and pancreatic amylase has previously been demonstrated in a rat model, where the glucose-stimulated insulin response was associated with an increase in the amylase component of the exocrine pancreas zymogen granules, while that of chymotrypsinogen and lipase were decreased [10]. Results of the current study and those of our previous study [16] suggest, that blood amylase might depress the required insulin response by indirectly participating in glucose assimilation. On the other hand, during the early stages of life, amylase might take part in the utilization of glucose independent of insulin. It is well known that porcine neonates are used as a model for studying the gastrointestinal system of premature babies [22]. Naturally born pigs have an immature gastrointestinal tract, which corresponds to the tract of preterm human infants due to its advanced maturation which occurs only during the last stage of gestation. Thus, the development of the gastrointestinal tract in newborn piglets continues after birth and its functional capacity might not be the same as that of mature animals. In a previous study from our lab (Pierzynowski et al., 1993) we reported the failure of plasma insulin levels to rise in preweaned pigs, despite the increase in blood glucose levels, suggesting an immature glucose-stimulated insulin response in young piglets [23]. Based on our previous data, we assume that amylase might regulate immature intestinal enterocytes which are able to take-up and metabolize glucose. Alternatively, the role of amylase in glucose assimilation might be a specific characteristic of the immature endocrine pancreas.

The increased plasma amylase levels observed in neonate piglets has also been observed in human infants [5]. Taking into account the absence of specific substrates for enzymatic amylolytic action, it is possible that blood amylase levels are to some degree responsible for the regulation of carbohydrate assimilation at that age. The ratio of plasma insulin to plasma amylase activitiy could be an important measure that is often overlooked.

The increased blood amylase levels observed following duodenal bypass surgery could also be associated with a commonly reported improvement in insulin sensitivity after bariatric surgery. High blood amylase levels are often related to increased pancreatic production of amylase and its’ subsequent secretion into the bloodstream. Previously, Reid et al. (1933) reported an increased blood amylase activity in diabetic patients receiving dietetic, not insulin treatment [1]. This finding is in support of our results, suggesting a negative correlation between blood insulin and blood amylase levels. Blood amylase levels gradually increased in our DJB pig model following surgery (Fig 1A). DJB surgery creates physiological digestive inefficiency, which by means of natural feedback mechanisms remains in the alimentary and common limbs, stimulating exocrine pancreatic function which can lead to increased plasma amylase activity. It is worth to notice that we suggest possible regulatory effects of blood amylase only on post-prandial hyperglycaemia and insulin response, not on the basal plasma levels of glucose and insulin. It is of great importance for the maintenance of homeostasis that basal levels are kept constant and thus some sort of regulation is needed in order to ensure a successful balance and a rapid return to euglycaemia.

The results, observed after intravenous infusion of amylase demonstrate that pancreatic amylase, in particular, is able to regulate the glucose-stimulated insulin response. The importance of amylase in circulation is evident, and in the case of exocrine pancreatic failure, the organism might try to normalise blood amylase levels, using other amylase sources. This could explain the unreliable and surprisingly high levels of serum/plasma amylase levels reported during exocrine pancreatic insufficiency [16].

In conclusion, our findings propose that the outflow of amylase from the pancreas to the interstitial blood participates in the regulation of preprandial and postprandial glucose-stimulated insulin responses. The results of the current study show an inverse relationship between blood amylase and blood insulin levels, suggesting an important role of blood amylase in the assimilation of dietary glucose in growing pigs.
